# Menstrual and sexual health education in Brazil's School Health Program: an experience report in medical education

**DOI:** 10.3389/fpubh.2026.1730562

**Published:** 2026-03-06

**Authors:** Zibeilde Ferreira Borges Paschoalini, Marcello da Silveira Paschoalini

**Affiliations:** Faculdade Sulamérica, Luís Eduardo Magalhães, Bahia, Brazil

**Keywords:** adolescent health, community-based learning, experiential learning, gender equity, medical education, menstrual health, School Health Program (SHP), service-learning

## Abstract

**Background:**

Menstrual health is a critical public health issue tied to gender equity and adolescent well-being. Brazil's School Health Program (SHP) mandates sexual and reproductive health education, yet structured models for integrating medical students into this policy-driven, community-based work are scarce. This study describes and analyzes an experiential learning project where first-year medical students facilitated menstrual health education within the SHP.

**Methods:**

A qualitative case study was conducted. Six female medical students developed and delivered a developmentally tailored educational session on puberty, menstrual physiology, and dignity to 5th-grade (elementary school) girls at a public school, as part of the 2025 activities of the SHP. Data from structured reflective debriefings with students, supervisor observations, and SHP records were analyzed through thematic content review and consensual validation.

**Results:**

Analysis revealed three primary learning outcomes for medical students: (1) Development of core communication and empathetic skills for discussing sensitive topics; (2) A deepened, experiential understanding of menstrual stigma and social determinants of health; and (3) Practical insights into intersectoral collaboration and public policy implementation through the SHP framework. School staff observed a marked reduction in embarrassment and increased openness to discussion among the adolescent participants.

**Conclusion:**

Integrating menstrual health promotion into early medical training via established public policy platforms like the SHP is feasible, low-cost, and highly effective. This model serves as a dual-purpose intervention, fostering socially accountable, humanistic competencies in future physicians while simultaneously advancing adolescent health literacy and dignity. It provides a replicable framework for community-engaged medical education in Brazil and similar low-resource settings.

## Introduction

Menstrual health is a cornerstone of adolescent well-being, gender equity, and the fulfillment of human rights (1, 2). Despite global recognition, significant gaps persist in menstrual literacy, with stigma, misinformation, and inadequate preparedness among educators continuing to undermine the health, dignity, and school participation of girls, particularly in socioeconomically vulnerable communities (3, 18, 20).

Concurrently, medical education is undergoing a paradigm shift toward greater social accountability and community engagement (4), calling for pedagogical models that place students in real-world contexts to develop core competencies (5). This shift is particularly relevant for addressing sensitive public health issues like menstrual health, **especially considering that gaps in reproductive and fertility-related knowledge have been documented even among medical trainees. Studies from high-income countries have shown that residents may enter clinical practice with limited understanding of core reproductive health concepts** **(****6**, **7****)**. This study acknowledges an important pedagogical consideration raised by literature in other settings: the need to ensure that medical students themselves are adequately prepared to address reproductive health topics. In direct response to this consideration, our experiential model incorporated a structured, faculty-supervised preparatory phase specifically designed to build foundational competence and confidence.

Prior to the community intervention, the six first-year medical student facilitators engaged in a multi-stage preparation. This involved: (1) guided research on national policy documents (SHP, Menstrual Dignity Program) and Brazilian epidemiological data on menstrual health; (2) collaborative development and iterative refinement of educational slides and interactive activities; and (3) participatory workshops and role-playing simulations, all under the close mentorship of a faculty member with extensive experience in the School Health Program. This scaffolded process ensured that the students not only grasped the biomedical and psychosocial dimensions of menstrual health but also practiced sensitive, age-appropriate communication in a supportive learning environment.

Therefore, this project presents a case study in which a potential educational gap—student preparedness on a sensitive topic—is explicitly addressed through curricular design. By transforming preliminary uncertainty into a structured learning objective, the model aims to fulfill a dual purpose: responding to a critical community health need while fostering early development of humanistic, communication, and public health competencies in future physicians. This approach is grounded in Kolb's (8) experiential learning cycle and service-learning frameworks (9), where deliberate preparation enables meaningful and effective community engagement.

In Brazil, this educational philosophy aligns seamlessly with national public policies. The intersectoral School Health Program (SHP) creates a formalized bridge between Family Health Strategy (FHS) units and public schools. The current cycle of the program (2025–2026) establishes various thematic axes, among which 'Sexual and Reproductive Health' is a key priority—a scope that inherently encompasses menstrual health education (10). This policy infrastructure presents a unique and underutilized opportunity for authentic, policy-aligned community-based medical education (CBME). **This initiative is also aligned with the World Health Organization's Health Promoting Schools framework, which emphasizes whole-school approaches to health that integrate education, health services, and community engagement. The WHO–UNESCO global standards highlight schools as strategic settings for health promotion, equity, and life-course well-being, particularly through intersectoral collaboration and age-appropriate health literacy interventions** **(****11****)**.

However, a significant gap persists at the intersection of these converging priorities: a documented scarcity of structured, pedagogically grounded models through which early-year medical students—who may themselves benefit from foundational reinforcement in menstrual health literacy—can lead educational interventions within formal policy structures like the SHP. This study addresses this multifaceted gap by presenting an experiential case study.

The School Health Program (SHP) is formally integrated into the medical curriculum as part of a Problem-Based Learning (PBL) approach to community-based education. Within this framework, menstrual and sexual health is a pre-established thematic axis, defined by public policy and institutional teaching plans. Accordingly, first-year medical students did not select the topic autonomously but were tasked with developing and implementing the practical component of this theme under faculty guidance.

The activity aimed to serve a dual purpose: enhancing the menstrual literacy and dignity of schoolgirls while providing medical students with an early experiential learning opportunity grounded in public policy, social accountability, and humanistic care. It describes the implementation and analyzes the outcomes of a menstrual health education session developed and facilitated by first-year medical students as an integral component of the SHP.

## Materials and methods

### Study design and pedagogical framework

This study employed a qualitative, single-case study design (12) to provide an in-depth exploration of an experiential educational intervention. The project was explicitly grounded in Kolb's (8) experiential learning theory and a service-learning model (9), where academic learning is applied to address genuine community-identified needs—in this case, the school's and SHP's mandate for adolescent sexual health education.

### Setting and policy context

The activity was conducted within the formal structure of the SHP in the municipality of Luís Eduardo Magalhães, Bahia. It was conducted in 2025 under the established pairing between the Yoshio Shirabe Family Health Strategy (FHS) unit and the Pedro Paulo Côrte Filho Municipal School. The “Sexual and Reproductive Health” axis was designated to our student group, providing a clear policy mandate for the menstrual health focus. The session content was further informed by the objectives of the federal Menstrual Dignity Program (13). This policy alignment is documented in Supplementary File 2.

### Participants

**Facilitators:** the six female facilitators were first-year medical students in a six-year undergraduate program. **Their estimated age range was 17–22 years. Most students entered the 6-year undergraduate medical program directly after completing secondary education, although a small proportion held prior undergraduate degrees (e.g., pharmacy or nutrition)**. A female faculty member and the FHS nurse coordinator provided supervision.

**Community participants:** The target group was all 5th-grade (elementary school) girls (ages 10–12, *n* = 53) at the partnered school. The school leadership specifically requested female-only facilitators to ensure a comfortable and private environment for the adolescents. This grade level was strategically selected because it encompasses the age range when menarche typically occurs or when early signs of puberty become evident. Girls at this developmental stage are often curious, have questions and concerns about their changing bodies, and are thus highly receptive to educational activities focused on health, self-care, and understanding bodily changes. The school environment provided a structured and safe setting for this targeted, sensitive, and age-appropriate intervention.

### Intervention design and implementation

Prior to the field intervention, the student facilitators participated in a structured problematization session based on Problem-Based Learning (PBL) principles (Supplementary File 4). In this preparatory meeting, the group reviewed the relevant SHP policy documents and discussed public policies related to the theme, including the Menstrual Dignity Program established by Federal Decree No. 11.432. These references served as the basis for understanding the institutional framework of menstrual health in the school context and its link to actions promoting equity and adolescent well-being. During this phase, priority needs related to menstrual health, menstrual dignity, and communication with 5th-grade girls were identified. The group discussed the sexual and reproductive health thematic axis outlined in the teaching plan and the SHP, as well as the anticipated challenges of addressing a sensitive topic in the school environment. This step preceded the development of educational materials and aimed to define how the topic would be approached with the adolescents, always with an educational focus, considering their developmental stage, age-appropriate language, potential discomfort, and cultural barriers. Collaborative pedagogical objectives were defined, focused on promoting welcome, active listening, and reducing menstruation-related stigma.

### Preparation of student facilitators

Beyond the initial problematization session, student preparation occurred in multiple stages. First, a theoretical briefing was conducted, focused on a general understanding of puberty, menarche, and menstrual care, always with an educational focus and aligned with SHP guidelines and actions promoting menstrual dignity. Subsequently, a specific workshop on sensitive language and communication was held to ensure the use of simple, clear, and age-appropriate terms for the adolescents, prioritizing comfort, understanding, and emotional safety. This stage involved discussing how to present the topic of menstruation in an accessible way, using examples close to the students' reality and avoiding overly technical approaches. **Although formal simulations with standardized patients were not used, the students participated in role-playing exercises among themselves, practicing dialogue facilitation, active listening, and responses to anticipated sensitive topics, with direct feedback from the supervising faculty**. Only after this pedagogical and communicational preparation were the final educational materials—including slides and interactive activities—developed by the students. These materials underwent iterative review and were approved by the supervising faculty for pedagogical suitability, age-appropriateness, and alignment with SHP guidelines before the field activity.

### Session structure and content

Informed by this planning, the facilitators—under close supervision—developed and delivered a 90-minute interactive session, structured into five sequential components:

1. Introduction and safe space setting: establishing rapport, group norms, confidentiality, and a supportive environment for open discussion.
2. Puberty and menarche dialogue: a conversation normalizing bodily changes, using inclusive and age-appropriate language.
3. Interactive physiology activity: a collaborative puzzle illustrating the key phases of the menstrual cycle in a simplified and engaging format.
4. Menstrual dignity and stigma reduction: a guided discussion to bust myths, address self-care and bodily autonomy, and counter culturally embedded misconceptions.
5. Anonymous Q&A: a “question box” that allowed the girls to submit queries anonymously, fostering psychological safety and enabling candid dialogue, with responses provided openly by the facilitators. This strategy was chosen due to the sensitive nature of the topic.

All educational materials and activities were vetted for age-appropriateness and were fully aligned with both national SHP guidelines and the pedagogical objectives established during the PBL-based planning phase.

### Data collection

Data for this experience report were derived from a structured, post-intervention pedagogical analysis aimed at capturing the medical students' learning. The primary source was a facilitated group debriefing session conducted immediately after the school activity. In this session, the six student facilitators, guided by the supervising faculty (Z.F.B.P.), engaged in a collective reflection on their experiences, challenges, and insights. The discussion was structured around key prompts related to communication, observed social determinants, and the experience of working within the SHP framework. The supervisor documented this discussion in comprehensive narrative notes. Second, the supervising faculty member recorded narrative observational notes during and immediately after the activity, documenting student facilitation processes, group dynamics, and general engagement levels. Third, official School Health Program (SHP) records, including municipal planning documents and activity logs, were consulted to confirm institutional alignment, implementation context, and policy integration. An anonymous question box was used exclusively as a pedagogical tool during the educational session to promote psychological safety and facilitate student learning in addressing sensitive topics. The content of the questions was not recorded, transcribed, categorized, or analyzed and therefore did not constitute a data source for this study. **All reflections refer exclusively to the medical students' learning process and to themes anticipated during preparatory training; no questions or statements from adolescent participants were recorded, transcribed, or analyzed**.

### Analytic approach

To synthesize the core learning outcomes from the experience, a thematic analysis of the reflective debriefing was conducted following established qualitative analysis procedures (19). The process was pedagogical and qualitative, focusing on the insights generated by the students themselves:

1. Documentation and familiarization: the supervising faculty's detailed notes from the debriefing session served as the primary data corpus.
2. Identification of key insights: the faculty researcher (Z.F.B.P.) reviewed the notes to identify salient and recurrent themes expressed by the students during the discussion (e.g., initial nervousness, moments of empathic connection, observations about stigma, understanding of policy implementation).
3. Consensual validation and theme formation: these initial themes were presented and discussed with the student facilitators in a subsequent meeting. Through this member-checking process, the students confirmed, refined, and named the themes that most accurately represented their shared learning. This collaborative, consensual approach ensured the results were grounded in the students' lived experience. The final thematic framework is presented in Table 1.

**Table 1:** Summary of themes, subthemes, illustrative excerpt from medical student reflection.

**Theme**	**Subthemes**	**Illustrative quote from student reflection**
Humanistic and communication competencies	Empathy in action; nuanced communication; creating safety	“I stopped being just a student with a lesson plan and started being a listener. Their comfort became my priority, and the learning flowed from that.”
Social determinants and menstrual equity	Confronting stigma; identifying structural barriers; professional agency	“It's one thing to read about period poverty. It's another to recognize how structural barriers shape the lived realities of girls in vulnerable contexts. That structural barrier has a face now.”
Intersectoral collaboration	Policy-to-Practice; collaborative workflows; school as health setting	“The SHP wasn't a vague government program anymore. It was the nurse, the teacher, the principal, and us, all in a room making this happen. I understand ‘SUS' differently now.”

### Ethical considerations

This project was conducted as a pedagogical activity integrated into the mandatory community-based curriculum of the medical program. In accordance with Brazilian Resolution CNS 510/2016, activities of this nature—which are part of professional training and do not involve the collection of personally identifiable data for research purposes—do not require formal review by a research ethics committee. All procedures adhered strictly to institutional educational guidelines and the principles of the Declaration of Helsinki. Formal authorization for the activity was obtained from the Municipal Health and Education Secretariats of Luís Eduardo Magalhães, the FHS Yoshio Shirabe unit, and the school principal (as formally documented in Supplementary File 1). The school confirmed that standard SHP parental authorization procedures, which cover educational health activities, were followed for all participating adolescents. The medical student participants provided consent for the educational use of their anonymized reflective insights as part of their course requirements. No images or any personally identifiable data from the schoolgirls were collected or recorded. No content produced by the adolescent participants, including verbal or written questions, was collected, recorded, or analyzed for research purposes.

## Results

### Synthesis of medical students' reflective learning

The consensual analysis of the students' reflective debriefing yielded three interconnected thematic categories that capture the essence of their experiential learning. These are summarized in Table 1.

### Pedagogical insights from an anonymous question box activity

The anonymous question box functioned as a formative educational device rather than a source of analyzable data. Its use enabled the medical students to practice responding to sensitive, age-appropriate health concerns in real time, thereby strengthening communication skills, emotional attunement, and adaptability. The variety of themes raised through this activity informed the students' reflective learning processes, without generating any recorded or research-analyzed data from the adolescent participants.

### Communication, empathy, and humanistic competencies

Students consistently described this as their first practical test in communicating complex, sensitive health information. The experience forced a rapid evolution from theoretical knowledge to applied skill.

**Empathy in action:** students reported moving beyond sympathy to a felt understanding of the girls' perspectives. One shared insight noted, “I saw their initial shyness and remembered my own confusion at that age. It wasn't about teaching at them, but connecting with them.”

**Nuanced communication:** they learned to calibrate language, tone, and non-verbal cues. A student reflected, “I realized technical terms like ‘endometrium' created distance. When I said ‘the nourishing lining inside the uterus,' their eyes showed understanding. Listening to their word choices became my guide.”

**Creating psychological safety:** the conscious decision for female-only facilitation, initially a logistical detail, was reflected upon as a critical intervention for trust-building.

**Understanding social determinants and menstrual equity:** the classroom encounter transformed abstract concepts like “stigma” and “vulnerability” into tangible realities.

**Confronting stigma directly:** student reflections were marked by revelations about the pervasiveness of shame. Students reported that the necessity to address anonymously submitted concerns challenged them to translate biomedical knowledge into reassuring, age-appropriate language while maintaining sensitivity to cultural and social contexts. **One student reflected on being challenged by questions related to widely recognized myths about menstruation and virginity—issues that had been anticipated during the preparatory training—underscoring how cultural silence and stigma shape adolescents' concerns in real-world educational settings**.

**Identifying structural barriers:** observations went beyond individual knowledge gaps. Students noted the lack of discrete bins in bathrooms and how girls strategized to hide sanitary products, linking these to broader issues of infrastructure and dignity.

**Recognizing professional agency:** This experience fostered a sense of responsibility. A student concluded, “As a future doctor, I can be a source of accurate information, but also an advocate for better school facilities and policies that don't force girls to hide.”

### Operationalizing intersectoral collaboration and public policy

The project demystified the workings of the **Brazilian Unified Health System (SUS)** and the SHP.

**From policy document to practice:** students gained appreciation for the planning behind community health. “I finally saw how the ‘Health at School' checkbox in our municipal plan translates into a real morning at a school, with a nurse, a teacher, and us all working from the same script,” one observed.

**Learning collaborative workflows:** the coordination with the FHS nurse and school teachers provided a practical lesson in interprofessional and intersectoral dynamics. They saw firsthand the negotiation of roles, resources, and schedules that underpins community health work.

**The school as a health promotion setting:** the activity cemented the concept of the school as a crucial space for primary prevention and health literacy, a core principle of the SHP that became experientially validated.

## Discussion

This experiential learning project demonstrates that integrating menstrual health education into the earliest stages of medical training is not only feasible but also a potent pedagogical strategy. By embedding the intervention within the mandated structure of Brazil's SHP, the project achieved dual alignment: with national public health priorities and with contemporary frameworks for socially accountable medical education (4). Our findings strongly support the educational value of such community-based experiences. The development of empathy and communication skills observed aligns with core objectives of humanistic medical education (14) and underscores the argument that these competencies are best cultivated through authentic human interaction, not simulated scenarios alone. Furthermore, students' visceral encounter with menstrual stigma and its social underpinnings provided a more profound understanding of gender equity and social determinants of health than traditional coursework typically allows. This echoes the transformative potential of service-learning to foster critical consciousness (15). The use of an anonymous question box as a core pedagogical strategy proved to be a significant factor in the session's dynamics. This tool, recommended for engaging adolescents on sensitive health topics (3), served a dual pedagogical function in our context. Primarily, it provided a safe channel for the schoolgirls to express concerns, thereby creating an authentic training ground for the medical students. The necessity to formulate clear, empathetic, and age-appropriate responses to spontaneous, real-world questions in real-time constituted a powerful exercise in adaptive communication and emotional attunement. This experiential challenge is aligned with findings from similar educational initiatives, such as the 'My Vital Cycles^®^' program in Australia, which underscores the value of interactive, student-centered methodologies for improving menstrual health literacy (16). In our model, the tool's primary and analyzed outcome was the competency development of the future physicians, reinforcing that creating psychologically safe environments is itself a critical clinical and educational skill. The project also served as a practical masterclass in health systems science. Students witnessed the operationalization of a national policy (the SHP), experiencing firsthand the complexities and rewards of intersectoral collaboration between health and education sectors. This early exposure is crucial for training future physicians who are not only clinically competent but also effective navigators and advocates within public health systems (17).

### Study strengths, limitations, and future directions

The strengths of this model include its direct alignment with national public policy, low operational cost, and reliance on existing public system infrastructure (the SHP and FHS), which underpin its scalability and replicability in other Brazilian municipalities and similar low-resource settings internationally. The positive reception from school staff and the observable reduction in embarrassment among the schoolgirls affirm its feasibility and immediate impact. This study has several limitations. As a qualitative case study, its findings, while rich in depth, are not generalizable. The voluntary nature of student participation may introduce selection bias, attracting those already interested in community health or gender equity. We lack longitudinal data to assess the sustained impact of the intervention on either the adolescents' health literacy or the medical students' professional development. Furthermore, parental consent covered the adolescents' participation in the educational activity but did not extend to the systematic collection or analysis of their specific questions for research purposes, which precludes a detailed content analysis of their queries. Future iterations of this model should incorporate structured pre- and post-intervention assessments of student attitudes and skills. Seeking participatory feedback from adolescent participants and their families could further ensure the intervention remains community-centered. Scaling the model to more schools, integrating male medical students in complementary educational roles (e.g., discussing puberty with boys to foster allyship), and conducting longitudinal assessments are promising future directions. The implementation of this model provided valuable operational and pedagogical insights. Initial considerations involved coordinating between health and education sectors and adapting to school schedules. A key process was supporting first-year medical students to navigate their initial hesitancy in discussing sensitive topics with adolescents. This was addressed through a structured preparatory phase: a theoretical briefing defined the core topics, and a dedicated session established appropriate, age-specific terminology. This preparation was crucial. Equipped with this clear framework and vetted language, the students' initial apprehension was transformed. During the intervention, their engagement became notably fluid and confident, demonstrating how targeted preparation can effectively build competence for community dialogue. This experience underscores that such foundational training is not just beneficial but essential for the success of experiential learning in sensitive domains. The model's core design features underpin its scalability: direct alignment with national public policy (the SHP), low operational cost, and reliance on existing infrastructure. This framework is readily adaptable to other Brazilian municipalities and offers a relevant template for similar settings internationally.

## Conclusion

This study presents a viable and impactful model for integrating menstrual health education into early medical training through Brazil's established School Health Program. The project successfully transformed a public health mandate into a rich experiential learning opportunity, demonstrating that such community-based activities can simultaneously advance adolescent health literacy and cultivate a generation of physicians who are empathetic, socially aware, and skilled in community engagement from the outset of their careers. By leveraging existing policy platforms, this approach provides a sustainable, scalable, and powerful pathway toward both menstrual equity and the development of socially accountable healthcare professionals.

## Data Availability

The original contributions presented in the study are included in the article/Supplementary material, further inquiries can be directed to the corresponding author.
