# Supplementary material for: Menstrual and sexual health education in Brazil's School Health Program: an experience report in medical education
Source: Front Public Health. 2026 Mar 6;14:1730562. doi: 10.3389/fpubh.2026.1730562 (PMC13002779; doi:10.3389/fpubh.2026.1730562)
Supplement: Supplementary file 1 [file Data_Sheet_1.pdf]

SUPPLEMENTARY FILE 1 — Institutional Authorization Letter

(Official English Translation + Original Portuguese Document)

PAGE 1 — Official English Translation

INSTITUTIONAL AUTHORIZATION LETTER

(Official English Translation for Academic Submission)
School Health Program (PSE)
Municipality of Luís Eduardo Magalhães — Bahia — Brazil

I, **Edineusa Souza Nascimento**, Principal of **Escola Municipal Pedro Paulo Côrte Filho**, hereby certify that:

On **May 29th, 2025**, this school participated in official actions of the **Brazilian School Health Program (Programa Saúde na Escola – PSE)** carried out by the **Family Health Strategy (ESF) Unit Yoshio Shirabe**, in partnership with **Faculdade Sulamérica – Medical Program**, Luís Eduardo Magalhães, Bahia, Brazil.

The activities included educational actions on **menstruation and the first menstrual cycle**, directed to **5th-grade girls**, in accordance with the **PSE 2025–2026 federal guidelines**.

Following the activity, the school team observed:

1. Greater comfort among students when discussing puberty and menstrual topics;
2. Reduction of taboos and embarrassment related to menstruation;
3. Strengthening of school–family–health sector integration;
4. Recognition of the school as a safe and health-promoting environment.

We further certify that:

- **No student was individually identified;**
- **No personal data or images were collected;**
- Participation occurred as part of routine PSE educational activities;
- The activity had an **exclusively pedagogical** character and did **not** constitute research;
- The school authorizes the academic description of this activity for scientific dissemination, provided that anonymity and confidentiality of all students are fully preserved.

Signed in Luís Eduardo Magalhães, Bahia, Brazil
December 11th, 2025

Signatures / Institutional Stamps

Edineusa Souza Nascimento
Principal — Escola Municipal Pedro Paulo Côrte Filho
INEP: 29471400

Sulamérica Faculdade

MEDICINA

Original Portuguese Document (Scan)

ESCOLA MUNICIPAL PEDRO PAULO CÔRTE FILHO
AV. SALVADOR Nº 2631, CIDADE UNIVERSITÁRIA.

DECLARAÇÃO

Eu, Edineusa Souza Nascimento, diretora da **Escola Municipal Pedro Paulo Côrte Filho**, município de Luís Eduardo Magalhães – BA, declaro para os devidos fins que:

No dia **29/05/2025**, esta unidade escolar participou das ações do **Programa Saúde na Escola (PSE)** desenvolvidas pela **Unidade de Saúde da Família Yoshio Shirabe**, em parceria com a **Faculdade Sulamérica**.

As atividades incluíram ações de educação em saúde relacionadas ao tema **menstruação e primeiro ciclo menstrual**, dirigidas às alunas do 5º ano do Ensino Fundamental, em conformidade com o Documento Orientador do PSE 2025–2026.

Após essa atividade, a equipe desta escola observou:

1. Maior conforto das alunas ao discutir puberdade e temas menstruais;
2. Redução de tabus e constrangimentos relacionados à menstruação;
3. Fortalecimento da integração escola–saúde–família;
4. Valorização da escola como espaço seguro e promotor de saúde.

Certificamos ainda que:

- Nenhuma estudante foi identificada individualmente;
- Não houve coleta de dados pessoais ou imagens;
- A participação ocorreu como parte das atividades escolares regulares;
- A ação teve caráter exclusivamente pedagógico, não configurando pesquisa;
- Autorizamos a descrição da atividade em publicação acadêmica, preservando o anonimato da comunidade escolar.

Por ser a expressão da verdade, firmo a presente.

Edineusa Souza Nasciment
Diretora
Matrícula Nº4025

Diretora

Carimbo institucional

Escola Municipal
Pedro Paulo Côrte Filho
INEP - 29471400

Data: 11 / 12 / 2025
