# Supplementary material for: Menstrual and sexual health education in Brazil's School Health Program: an experience report in medical education
Source: Front Public Health. 2026 Mar 6;14:1730562. doi: 10.3389/fpubh.2026.1730562 (PMC13002779; doi:10.3389/fpubh.2026.1730562)
Supplement: Supplementary file 2 [file Data_Sheet_2.pdf]

SUPPLEMENTARY FILE 2 — PSE OFFICIAL EXTRACT

Brazilian School Health Program (Programa Saúde na Escola – PSE)
Official Guidelines Relevant to the Menstrual Health Educational Activity

1. Federal PSE Guideline Extract (2025–2026 Cycle)

(Public-domain policy document issued by the Brazilian Ministry of Health)

The menstrual health educational session delivered in this study was aligned with the **PSE 2025–2026 federal guidelines**, as defined in:

Ministério da Saúde. Nota Técnica nº 30/2024 – CGEDESS/DEPPROS/SAPS/MS, which establishes the thematic axes and operational guidance for School Health Program actions nationwide.

Relevant Extract — Thematic Axis

For the 2025–2026 cycle, all municipalities must implement actions within the following mandatory thematic areas:

- **"Saúde Sexual e Reprodutiva" (Sexual and Reproductive Health)** — including menstrual health, puberty, first menstrual cycle, and adolescent reproductive health literacy.

Relevance to the Study

The intervention described in the manuscript falls directly under this federal axis, fulfilling required educational actions for public schools under national PSE policy.

**2. Municipal Assignment of Thematic Axes

(Luís Eduardo Magalhães – Bahia)**

(Confirms that the participating ESF unit is officially assigned to deliver this thematic axis)

The Municipality of Luís Eduardo Magalhães publicly assigns thematic responsibilities to each Family Health Strategy (ESF) team as part of the PSE cycle.

The **ESF Yoshio Shirabe Unit**, which implemented the activity in this study, is officially assigned to the thematic axis:

"Saúde Sexual e Reprodutiva — Menstrual Health Education and Puberty Guidance."

This assignment appears in:

- **Documento Orientador do PSE – Ciclo 2025–2026**
- **Municipal Table of Thematic Axes by ESF Units**

Both are public documents.

Relevant Extract (Paraphrased)

- **Unit:** ESF Yoshio Shirabe
- **School:** Escola Municipal Pedro Paulo Côrte Filho
- **Assigned Axis:** Sexual and Reproductive Health
- **Specific Action:** Menstrual health education for 5th-grade girls
- **Cycle:** 2025–2026

This demonstrates that the intervention was not exceptional or researcher-driven, but a **routine action mandated by federal and municipal PSE guidelines**.

3. Compliance and Alignment Statement

The menstrual health educational session described in the manuscript was implemented:

✓ In full compliance with federal PSE guidelines (Nota Técnica nº 30/2024)
✓ In alignment with the municipal assignment for the ESF Yoshio Shirabe Unit
✓ As part of routine school health activities
✓ Without collecting personal or identifiable data
✓ Fully aligned with national health promotion and adolescent health priorities

4. Files Attached to This Supplement (If Requested by Editors)

(Not included here to avoid uploading large government PDFs)

- **Federal Extract:** Nota Técnica nº 30/2024 (public-domain document)
- **Municipal Extract:** Table of thematic assignments (public document)

No student-level or confidential data is included in these documents.
