# Supplementary material for: Menstrual and sexual health education in Brazil's School Health Program: an experience report in medical education
Source: Front Public Health. 2026 Mar 6;14:1730562. doi: 10.3389/fpubh.2026.1730562 (PMC13002779; doi:10.3389/fpubh.2026.1730562)
Supplement: Supplementary file 3 [file Data_Sheet_3.pdf]

Supplementary File 3 — CODING FRAMEWORK

(Analytic Appendix – Qualitative Descriptive Content Analysis)

This supplementary file provides a transparent description of the analytic procedures used in the study, including the coding framework, theme definitions, and an illustration of how raw textual observations were transformed into codes, subthemes, and overarching themes. No participant-identifiable data are included.

1. Overview of Analytic Approach

A descriptive content analysis was conducted following established qualitative methodologies (Miles et al., 2014). The analytic steps included:

- **Familiarization:** Two coders independently read all reflexive student journals and supervisor observation notes.
- **Inductive Coding:** Each coder generated initial descriptive codes without a priori categories.
- **Code Comparison and Consensus:** Coders met for iterative reconciliation until consensus was reached.
- **Subtheme Development:** Related codes were grouped into conceptual subthemes.
- **Theme Construction:** Subthemes were integrated into higher-order themes.
- **Validation:** Cross-checking with raw materials, member checking with student facilitators, and triangulation with PSE documentation.

2. Coding Framework and Analytic Progression

The tables below illustrate the progression from initial codes to final themes.

Table S3.1 – From Initial Codes to Final Themes

Initial Codes (Inductive)	Subtheme	Final Theme (as in manuscript)
Nervousness / fear of error	Building Facilitator Confidence	**Communication, Empathy, and Humanistic Competencies**
Value of preparatory session		

Initial Codes (Inductive)	Subtheme	Final Theme (as in manuscript)
Observing embarrassment	Navigating Sensitive Conversations	
Creating safe environment		
Surprise at basic myths	Encountering Menstrual Stigma	**Understanding Social Determinants and Menstrual Equity**
Identifying structural barriers	Recognizing Social Determinants	
Learning about ESF-school collaboration	Understanding PSE Operationalization	**Operationalizing Intersectoral Collaboration and Public Policy**
Seeing policy in practice	Applied Policy Literacy	

3. Definitions of Final Themes
(Aligning exactly with the manuscript section "Results")

- **Theme 1: Communication, Empathy, and Humanistic Competencies** Progression from initial apprehension to growing comfort and self-efficacy when facilitating sensitive health topics with adolescents. Represents applied communication skill-building and experiential learning.
- **Theme 2: Understanding Social Determinants and Menstrual Equity** Recognition of taboos, myths, and structural barriers affecting menstrual health; emphasis on promoting dignity, accurate information, and safe spaces.
- **Theme 3: Operationalizing Intersectoral Collaboration and Public Policy** Insight into how national/local policy (PSE) is operationalized through collaborative workflows between ESF teams, schools, and university actors.

4. Illustrative Quotes from Student Reflections (Anonymized)

The following table presents direct, anonymized excerpts from student reflective journals, as explicitly requested.

Table S3.2 – Illustrative Quotes from Data Analysis

Final Theme	Initial Code	Direct Quote (Anonymized)
**Communication, Empathy, and Humanistic Competencies**	Fear of “saying something wrong”	*"Before starting, I was very nervous. What if I use a wrong word and embarrass one of the girls? But the preparatory meeting gave me a script to follow, which helped a lot."*
**Understanding Social Determinants and Menstrual Equity**	Surprise at myths	*"One girl asked, very seriously, if she could take a bath during her period. It was a shock to realize that such a basic myth was still present. We explained gently, and you could see her relief."*
**Operationalizing Intersectoral Collaboration and Public Policy**	Learning about PSE structure	*"I had no idea the nurse from the health center and the school teacher planned this together months ago. Seeing the PSE in action showed me how public policy actually reaches people."*
**Communication, Empathy, and Humanistic Competencies**	Value of safe environment	*"At first my hands were shaking when I spoke; by the end I was answering questions without checking my notes. Creating a safe space helped them and me."*
**Understanding Social Determinants and Menstrual Equity**	Identifying structural barriers	*"A girl whispered that she kept pads in her sock because she felt ashamed. That image changed how I see health access — it's not just about information."*

5. Reliability and Validation Procedures

- Two independent coders conducted the analysis.
- Consensus was reached through iterative discussion.
- Thematic interpretations were validated via member-checking with the student facilitators.
- Findings were triangulated with supervisor notes and PSE documentation.
