# Supplementary material for: Menstrual and sexual health education in Brazil's School Health Program: an experience report in medical education
Source: Front Public Health. 2026 Mar 6;14:1730562. doi: 10.3389/fpubh.2026.1730562 (PMC13002779; doi:10.3389/fpubh.2026.1730562)
Supplement: Supplementary file 4 [file Data_Sheet_4.pdf]

Supplementary File 4 — PBL Planning Protocol - Pedagogical Methodology Appendix — Problem-Based Learning

1. Context and Purpose

This document describes the preparatory pedagogical process used to design the menstrual health educational session implemented within the School Health Program (PSE). The preparation followed the Problem-Based Learning (PBL) methodology adopted by Faculdade Sulamérica, ensuring that students actively integrated theoretical foundations with community-based practice.

The activity was conducted as part of the Collective Health / School Health Practice component of the first-year curriculum, aligned with the following scheduled learning activities:

- **AULA 16 – 06/05/2025:** *Programa Saúde na Escola (PSE): Campo de prática*
  – Introduction to PSE policy
  – Discussion of roles of ESF, school, and intersectoral collaboration
  – Preparation for field activity (theory)
- **AULA 18 – 29/05/2025:** *Atividade de Promoção à Saúde na Escola: Campo de Prática*
  – Implementation of the educational session in the school
  – Supervised reflection and observation (practice)

(Note: AULA 17 was an exam and is not part of the pedagogical process for this intervention.)

2. The Guiding Problem ("The PBL Case")

Instead of a clinical scenario, students were presented with a community health and educational challenge, framed exactly as a PBL problem:

"As the ESF team responsible for the 'Sexual and Reproductive Health' axis of the 2025–2026 PSE cycle, how will your group design and deliver a 90-minute menstrual health educational session for 5th-grade girls that is developmentally appropriate, reduces stigma, and aligns with official Brazilian policy?"

This scenario required integrating scientific knowledge, communication skills, adolescent development principles, and public policy understanding.

3. Documents and Inputs Reviewed (Self-Directed Study)

Students examined:

- **Federal Policy:** *Nota Técnica nº 30/2024 — CGEDESS/DEPPROS/SAPS/MS* – Defines mandatory thematic axes for the PSE cycle, including Sexual and Reproductive Health and menstrual education.

- **Municipal Policy:** *Documento Orientador do PSE — Ciclo 2025–2026* – Includes the table assigning the "Sexual and Reproductive Health" axis to the ESF Yoshio Shirabe Unit and the partnering school.
- **Pedagogical and Scientific References:** Adolescent development (10–12 years), menstrual physiology education, communication strategies for sensitive topics, WHO and UNESCO recommendations on menstrual health, and the PBL methodology from Faculdade Sulamérica (Metodologia Ativa).

These resources informed the hypotheses, learning needs, and design decisions.

4. PBL Cycle Applied to the Planning Session

- **Phase 1 — Initial Group Discussion & Hypothesis Generation:** Students discussed anticipated fears among girls (shame, myths), challenges in presenting physiology simply, cultural stigma, limits of medical language, and how to create a safe space. They identified learning gaps and formulated learning objectives.
- **Phase 2 — Self-Directed Learning:** Between meetings, each student independently researched puberty development stages, menstrual cycle explanation appropriate for children, strategies to counter stigma and dismantle myths, how to structure anonymous Q&A for sensitive topics, and principles of inclusive health communication.
- **Phase 3 — Synthesis & Collaborative Solution Design:** During the synthesis meeting, the group shared their research findings, built a sequence of pedagogical activities, defined key messages in accessible language, selected interactive strategies (e.g., menstrual cycle puzzle), created guidelines for respectful myth-busting, and assigned facilitation roles among team members.

5. Final Output: Structured Educational Protocol (90 Minutes)

- **Introduction & Safe Space Setting (10 minutes):** Establish confidentiality and normalize the topic.
- **Puberty and Menarche Dialogue (15 minutes):** Age-appropriate explanations using inclusive, non-judgmental language.
- **Interactive Physiology Activity (20 minutes):** Puzzle-based menstrual cycle activity.
- **Menstrual Dignity & Stigma Reduction (20 minutes):** Guided myth-busting and discussion of self-care practices.
- **Anonymous Q&A (25 minutes):** Question box system with sensitive and supportive answers.

All materials used were aligned with Federal and Municipal PSE Guidelines and validated by the supervising faculty.

6. Alignment with Curriculum and Competency Development

This preparatory PBL cycle supported the development of: communication skills with children, health education competencies, understanding of intersectoral public health (PSE), professional identity formation, and the integration of theory and practice, as expected in Collective Health early training.

7. Reference

Faculdade Sulamérica. (n.d.). *Metodologia Ativa (PBL): Aprendizado Baseado em Problemas com casos clínicos*. Retrieved from https://sulamericafaculdade.com.br
